# Benign and malignant lesions of the mandible and maxilla: imaging findings and systematic approach

**DOI:** 10.1590/0100-3984.2025.0066

**Published:** 2026-04-23

**Authors:** Leonor Garbin Savarese, Murilo Bicudo Cintra, Marcello Henrique Nogueira-Barbosa, Gustavo Santos Boasquevisque

**Affiliations:** 1 Department of Medical Imaging, Hematology, and Clinical Oncology, Faculdade de Medicina de Ribeirão Preto da Universidade de São Paulo, Ribeirão Preto, SP, Brazil.; 2 Instituto do Câncer do Estado de São Paulo (ICESP), São Paulo, SP, Brazil.

**Keywords:** Mandible, Magnetic resonance imaging, Tomography, X-ray computed, Maxilla, Mandíbula, Imageamento por ressonância magnética, Tomografia computadorizada por raios X, Maxila

## Abstract

Although lesions of the mandible and maxilla are relatively common findings on imaging examinations, they still pose a diagnostic challenge for radiologists in clinical practice. Many of these lesions share similar radiographic characteristics, making it difficult to differentiate between benign, infectious, and malignant entities, which can delay appropriate management. This article aims to illustrate the main imaging findings and present a systematic approach based on radiological patterns, such as attenuation type, margins, relationship with dental structures, and anatomical distribution, in order to facilitate the differential diagnosis, as well as to guide clinical and therapeutic management.

## Abstract (pt)

As lesões ósseas da mandíbula e da maxila são achados relativamente frequentes em exames de imagem, mas ainda representam um desafio diagnóstico para o radiologista na prática clínica. Muitas dessas lesões compartilham características radiográficas semelhantes, dificultando a diferenciação entre entidades benignas, infecciosas e malignas, o que pode retardar a conduta adequada. Este artigo tem como objetivo ilustrar os principais achados de imagem e apresentar uma abordagem sistemática baseada em padrões radiológicos como tipo de atenuação, margens, relação com estruturas dentárias e distribuição anatômica, a fim de auxiliar na construção do diagnóstico diferencial e no direcionamento clínico-terapêutico.

## INTRODUCTION

Lesions of the mandible and maxilla encompass a variety of benign, infectious, fibro-osseous, and malignant entities, often with overlapping imaging characteristics, which makes the differential diagnosis difficult^**(1–^3^)**^. The correct identification of these lesions depends on a systematic analysis of their location, relationship with dental structures, margins, pattern of attenuation, and expansile behavior^**(4–^6^)**^. In clinical practice, the most widely used screening method is panoramic dental radiography, which is often responsible for the initial detection of these lesions. However, the use of computed tomography (CT) and magnetic resonance imaging (MRI) plays a fundamental role in the characterization of such lesions, allowing the evaluation of signs of aggressiveness, such as cortical rupture, soft-tissue invasion, and necrosis^**(2,^7^)**^. In addition, recent updates from the World Health Organization (WHO), such as the 2022 classification, have brought important changes in the approach to odontogenic tumors and fibro-osseous lesions^**(8)**^.

The use of CT and MRI plays a fundamental role in characterizing lesions of the mandible and maxilla, allowing the evaluation of signs of aggressiveness, such as cortical rupture, soft-tissue invasion, and necrosis^**(2,^7^)**^. In addition, recent WHO updates, such as the 2022 classification, have brought important changes to the approach to odontogenic tumors and fibro-osseous lesions^**(8)**^. This article aims to illustrate the main imaging findings of benign and malignant lesions of the mandible and maxilla, highlighting the radiological criteria that are useful in constructing the differential diagnosis.

### Odontogenic lesions

#### Radicular cyst

A radicular cyst, also known as a periapical cyst, is the most common inflammatory odontogenic cyst and a common sequela of pulp necrosis, resulting from a chronic inflammatory process (infection), usually secondary to dental caries or trauma. The infection extends to the apex of the tooth, leading to formation of the cyst.

**Figure 1:**
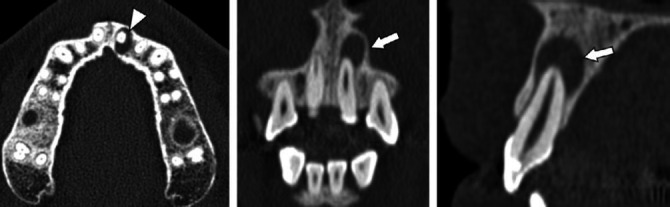
Radicular cyst in a 37-year-old female patient. CT images showing a well-circumscribed radiolucent lesion around the root of the left upper central incisor.

Figure 1 shows CT scans of a radicular cyst that appears as a well-defined, unilocular radiolucent lesion with fine cortical contours, located in the periapical region. Such cysts are typically less than 1 cm in diameter, and discrete bone expansion or fusion with contiguous lesions can occur^**(1,^3^)**^.

#### Dentigerous cyst

**Figure 2:**
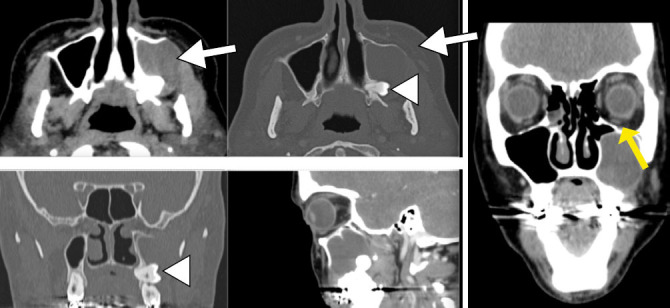
Dentigerous cyst in a 44-year-old female patient. CT scans showing a unilocular expansile radiolucent lesion around the crown of the impacted left maxillary third molar (arrowhead), with well-defined margins, extending to the left maxillary sinus and causing bone lysis in the posterolateral wall of the sinus (white arrows). Note the elevation of the left maxillary alveolar process, represented by a thin bony line (yellow arrow), indicative of growth of the lesion toward the maxillary sinus.

A dentigerous cyst, also known as a follicular cyst, is the most common developmental odontogenic cyst, associated with unerupted teeth, especially third molars and canines. It involves the crown of the tooth, with a predilection for young adults. It can reach large dimensions, promoting bone remodeling or displacement of adjacent teeth. On CT, it presents as a well-defined, unilocular radiolucent lesion involving the neck and crown of the tooth, with continuous cortical margins (Figure 2). Larger dentigerous cysts can extend to the maxillary sinus or mandibular ramus^**(1,^3^)**^.

#### Odontogenic keratocyst

**Figure 3:**
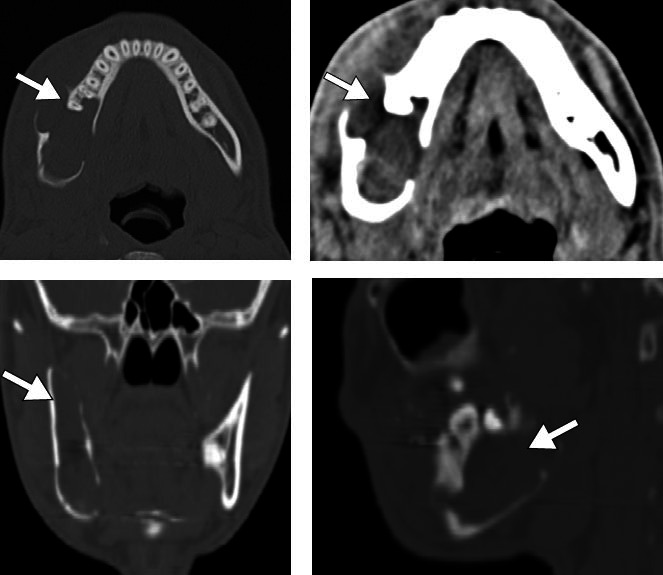
Odontogenic keratocyst in a 22-year-old male patient. CT images showing a radiolucent lesion in the right mandible, with thin, sclerotic borders, causing bone expansion and erosion, with resorption of tooth roots.

An odontogenic keratocyst is a benign but locally aggressive lesion derived from the dental lamina. With a predilection for the posterior mandible, it is characterized by infiltrative growth, a high recurrence rate, and the possibility of multiple lesions when related to Gorlin–Goltz syndrome. On imaging, it typically appears as a unilocular or multilocular radiolucent lesion with well-defined margins, frequently with discrete bone expansion and root resorption, with greater growth in the anteroposterior direction of the mandible (i.e., on the longitudinal axis), as shown in Figure 3. It presents with liquid or keratin content^**(8)**^, and MRI shows a cystic lesion without solid components and with peripheral enhancement.

#### Ameloblastoma

**Figure 4:**
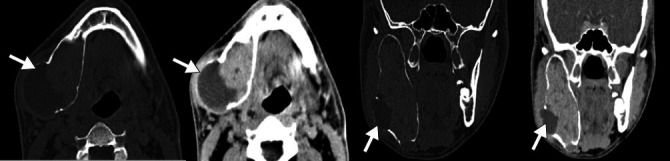
Ameloblastoma of the right mandible in a 25-year-old male patient. Axial and coronal CT images showing a radiolucent, solid–cystic, unilocular expansile lesion in the right mandible, with cortical thinning and destruction.

An ameloblastoma is a benign, yet aggressive, epithelial odontogenic tumor derived from enamelforming cells. It mainly affects young adults and is preferentially located in the posterior region of the mandible, frequently associated with impacted molars. Radiographically, it presents as an expansile lesion, typically multilocular with a classic soap-bubble or honeycomb pattern, and more rarely unilocular. Ameloblastomas typically show greater growth in the buccolingual direction of the mandible (i.e., along the transverse axis); there can also be resorption of adjacent roots and cortical thinning, with no periosteal reaction^**(2)**^. The solid portions of the lesion are most easily identified on MRI, on which they show restricted diffusion and contrast enhancement (Figure 4).

#### Ossifying fibroma

**Figure 5:**
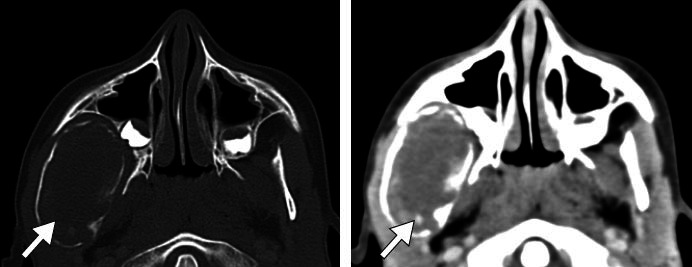
Ossifying fibroma in an 8-year-old male patient. CT scans showing an oval, predominantly radiolucent, lesion in the right ramus of the mandible, causing bone expansion and remodeling, as well as discrete foci of mineralized bone matrix (arrows).

Ossifying fibroma is a benign fibro-osseous lesion composed of fibrocellular stroma and variable mineralized material of periodontal origin. It mainly affects young women, between the second and fourth decades of life, and has a predilection for the posterior mandible. On imaging, it manifests as a well-defined unilocular lesion, with sclerotic margins, and can have a lytic, sclerotic, or ground-glass appearance depending on the evolutionary stage. Bone expansion and remodeling can occur (Figure 5). The juvenile variant presents with more aggressive behavior^**(9)**^.

#### Odontoma

**Figure 6:**
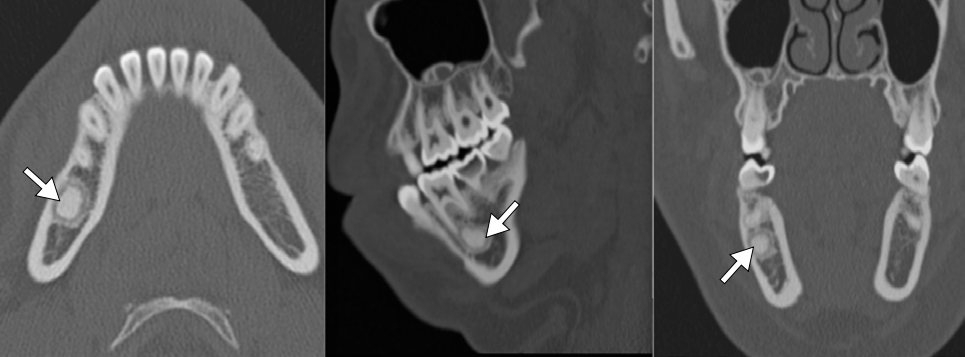
A 21-year-old female patient. CT scans showing a lesion with sclerotic borders, low-attenuation halo, and radiodense interior, with density similar to teeth, located in the right mandible next to the root of the first molar, consistent with odontoma.

Odontomas, categorized as hamartomas, are the most common odontogenic tumors and result from a developmental anomaly. They preferentially affect children or adolescents and can obstruct tooth eruption. Radiographically, they are well-defined lesions with sclerotic margins and a halo of low attenuation. They can be radiopaque, mixed, or (initially) radiolucent. They are classified as compound (with a tooth-like structure) or complex (a disorganized mass of dental tissue). The presence of the halo, as depicted in Figure 6, helps differentiate an odontoma from an osteoma^**(3,^6^)**^.

#### Florid cemento-osseous dysplasia

**Figure 7:**
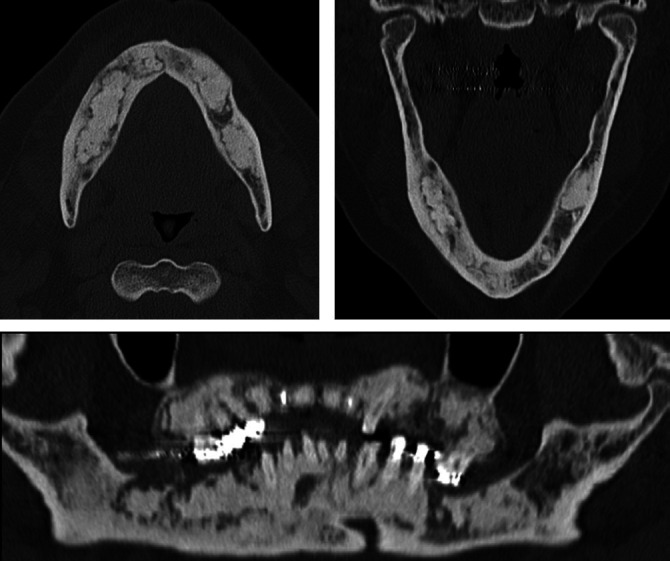
CT scans of a 50-year-old female patient, showing multiple well-defined radiodense areas, with a ground-glass appearance, diffusely occupying the periapical region in the mandible and maxilla, with an expansile effect, consistent with florid cemento-osseous dysplasia.

Florid cemento-osseous dysplasia is a benign fibro-osseous condition^**(3)**^, of hamartomatous nature, which typically affects two or more quadrants of the mandible and may involve the entire lower arch. Initially, it presents as radiolucent lesions, subsequently presenting as areas of ground-glass, radiopaque, or mixed attenuation. The margins are typically well defined. It is associated with an increased risk of osteomyelitis, especially after extractions or invasive procedures. The absence of tooth displacement helps differentiate florid cemento-osseous dysplasia from other expansile processes (Figure 7).

### Non-odontogenic lesions

#### Aneurysmal bone cyst

**Figure 8:**
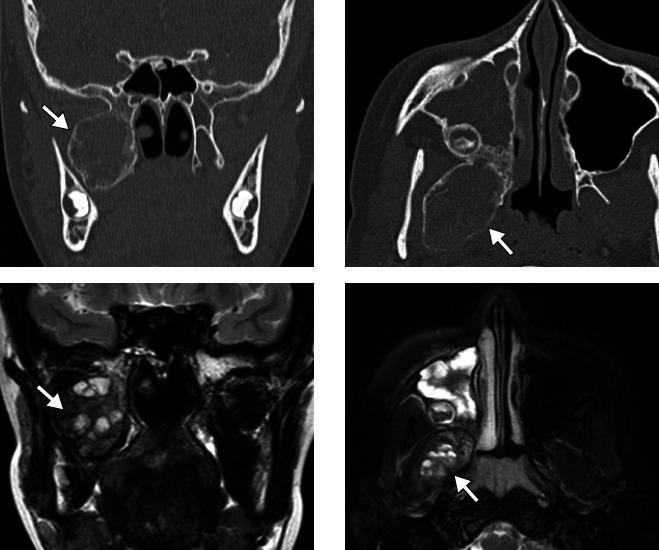
Aneurysmal bone cyst in a 13-year-old male patient. MRI scans showing a heterogeneous expansile formation centered at the right pterygomaxillary junction, showing areas of hyperintense signal on T2-weighted images that form levels within it, displacing the pterygoid muscles and the parapharyngeal space.

An aneurysmal bone cyst is a rare, non-neoplastic, expansile lesion of the mandible and maxilla. It is more common in young patients and can be associated with other lesions, such as ossifying fibroma and fibrous dysplasia. Radiographically, it presents as a unilocular or multilocular osteolytic lesion with multiple cystic cavities separated by septa, often with fluid-fluid levels identifiable on CT or MRI. The septa usually show enhancement after contrast administration^**(2)**^, as illustrated in Figure 8.

#### Central giant cell granuloma

**Figure 9:**
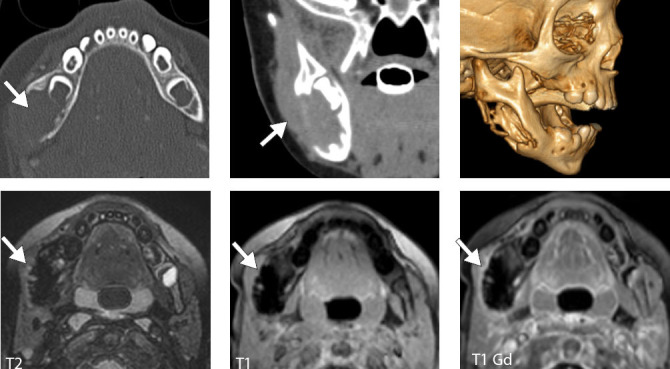
Central giant cell granuloma in a 16-month-old male patient. CT and MRI scans showing a cystic lesion in the right mandible, with expansion, remodeling, and bone erosion.

Central giant cell granuloma is a benign bone lesion caused by a reparative reaction to inflammation, possibly related to trauma. It typically affects girls and young women. Initially, it manifests as a small unilocular lesion; as it evolves, it can become multilocular and expansile, with cortical remodeling and erosion (Figure 9). The main differential diagnosis is with a brown tumor of hyperparathyroidism, with laboratory data and age range being fundamental for making that distinction^**(5)**^.

#### Osteoma

**Figure 10:**
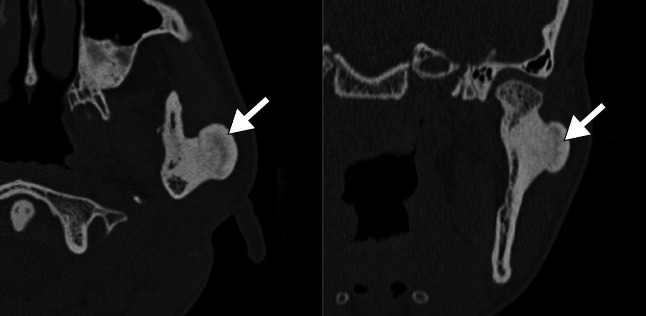
Osteoma in a 19-year-old female patient with Gardner’s syndrome. CT imag-es showing an osteoblastic lesion with exostosis in the left mandibular ramus, with well-defined margins.

Osteomas are benign bone tumors^**(10)**^ composed of mature compact bone. They typically affect the craniofacial bones, especially the posterior body and condyle of the mandible. They present as well-defined, usually pedunculated, sclerotic masses, not associated with teeth. There is no perilesional halo. There can be slight bone expansion. Osteomas are occasionally associated with simple bone cysts (Figure 10). A finding of multiple osteomas in the mandible should raise suspicion of Gardner’s syndrome.

#### Mandibular torus

**Figure 11:**
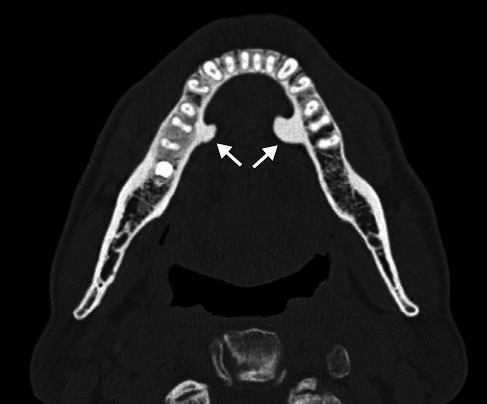
Mandibular torus in a 68-year-old male patient. Axial CT image showing bony protuberances (arrows) on the internal aspects of the mandibular bodies in the premolar region.

Tori are benign exostoses^**(6)**^ composed of dense cortical bone. They represent a common anatomical variant in adults, with a multifactorial etiology. They are generally asymptomatic, slow-growing, and covered by thin, poorly vascularized mucosa. They can be found in the lingual region of the mandible (mandibular torus), in the hard palate (palatine torus), or in the maxilla. Mandibular tori are well-defined lesions, with homogeneous bone attenuation and no peripheral halo (Figure 11).

#### Brown tumors

**Figure 12:**
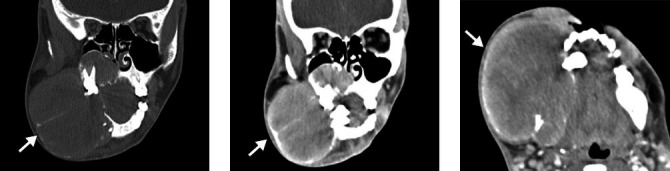
Brown tumor in a 21-year-old male patient. CT scans showing an extensive expansile lytic lesion with an inflated appearance, affecting the angle and body of the mandible on the right, with soft-tissue density and without enhancement after contrast administration. A similar lesion, affecting the maxillary and palatine bones, can be seen on the right, extending to the maxillary sinus, with destruction of its walls and invasion of the ipsilateral nasal cavity.

A brown tumor is a giant cell bone lesion^**(5)**^ secondary to long-standing hyperparathyroidism. It can affect any bone in the body, including the mandible and facial skeleton. Radiographically, it presents as a unilocular or multilocular osteolytic lesion, with well-defined or poorly defined margins, and can cause cortical expansion (Figure 12). Associated generalized bone demin-eralization is common. The diagnosis is confirmed by laboratory tests showing elevated parathyroid hormone and hypercalcemia.

#### Fibrous dysplasia

**Figure 13:**
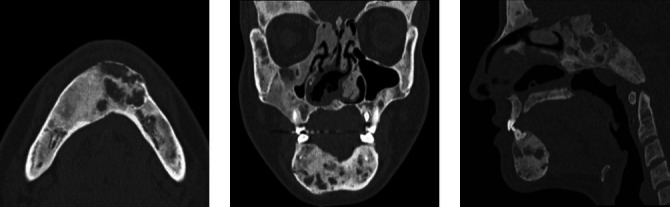
CT scans of a 20-year-old female patient, showing marked bone thickening and sclerosis, with a ground-glass appearance, involving the mandible, together with the frontal, ethmoid, sphenoid, and maxillary bones, as well as the nasal turbinates, all of which are consistent with polyostotic fibrous dysplasia.

Fibrous dysplasia is a benign bone lesion^**(9)**^ characterized by the replacement of normal bone with fibrous tissue and immature bone trabeculae. It can be monostotic or polyostotic, with polyostotic involvement being common in the head and neck due to the contiguity between the bones. Radiographically, it manifests as a lesion with poorly defined margins and ground-glass attenuation, although the pattern can be lytic, sclerotic, or mixed. It exhibits a longitudinal growth pattern, usually without tooth displacement (Figure 13). The differentiation between fibrous dysplasia and ossifying fibroma can be made on the basis of the poorly defined transition zone and greater infiltrative behavior of the former.

#### Osteoradionecrosis

**Figure 14:**
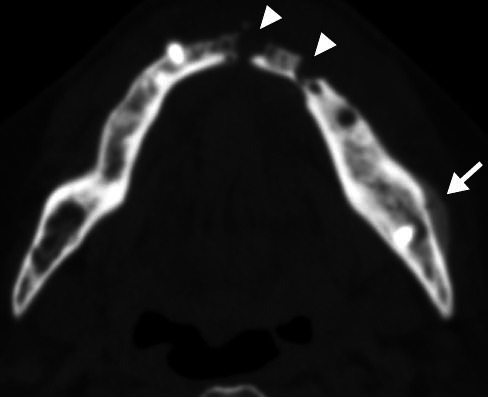
Osteoradionecrosis in a 67-year-old male patient with a history of radiotherapy. Axial CT image showing sclerosis, loss of bone trabeculae, cortical disruption, and an area of soft-tissue attenuation (arrow) in the mandible.

Osteoradionecrosis is a late complication of radio-therapy^**(7)**^ and can occur months to years after treatment. Its occurrence is related to the dose and duration of exposure. It preferentially affects the mandible, especially the mandibular body, due to reduced vascular supply. Radiographically, areas of sclerosis are observed interspersed with lytic regions, with irregular margins, cortical interruption, and bone sequestra (Figure 14). The main complication of osteoradionecrosis is osteomyelitis.

#### Osteomyelitis

Mandibular osteomyelitis is a bone infection^**(7)**^ usually associated with a history of immunosuppression, trauma, surgery, or radiotherapy. It can also develop as a complication of untreated dental infections.

**Figure 15:**
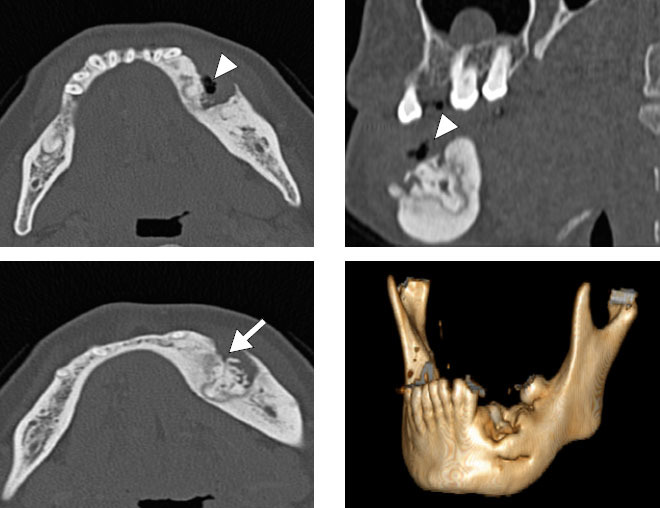
A 53-year-old male patient with mandibular osteomyelitis. Axial CT image with bone window settings showing a sclerotic sequestrum with a surrounding area of low attenuation (arrows).

In the acute phase, mandibular osteomyelitis may not present with imaging findings. In the chronic phase, cortical destruction, periosteal reaction, bone sequestra, obliteration of fat planes are seen, with a lytic, sclerotic, or mixed pattern (Figure 15). For assessing the extent of the lesion and the response to treatment, MRI can be useful.

#### Multiple myeloma

**Figure 16:**
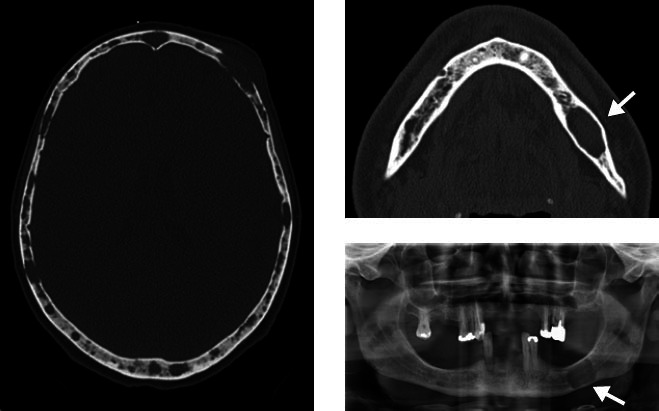
CT scans and panoramic radiograph of a 50-year-old female patient, showing multiple osteolytic lesions scattered throughout the skull, with well-defined margins and no reactive bone. Note the osteolytic lesion, without sclerotic margins, in the left mandible.

Multiple myeloma can affect the mandible in isolation or in the context of systemic involvement. The lesions may be asymptomatic and can, in some cases, precede the systemic diagnosis. Radiographically, multiple myeloma presents as well-defined ovoid radiolucent lesions with cortical erosion, without expansion or periosteal reaction (Figure 16). The typical “punched-out” lesions may be present, but are not mandatory for the diagnosis^**(3)**^.

### Malignant lesions

**Figure 17:**
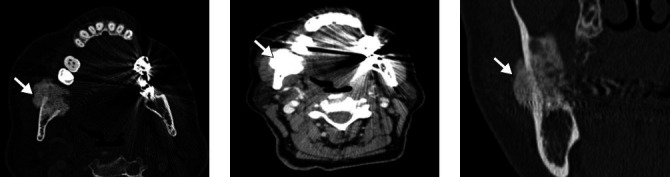
CT scans of a 57-year-old female patient, showing a lobulated exophytic cortical lesion at the angle of the right mandible, with a attenuation coefficient indicative of calcification, sclerosis (arrow), an irregular periosteal reaction, and a discrete soft-tissue component, consistent with neoplasia. The final diagnosis was osteosarcoma.

**Figure 18:**
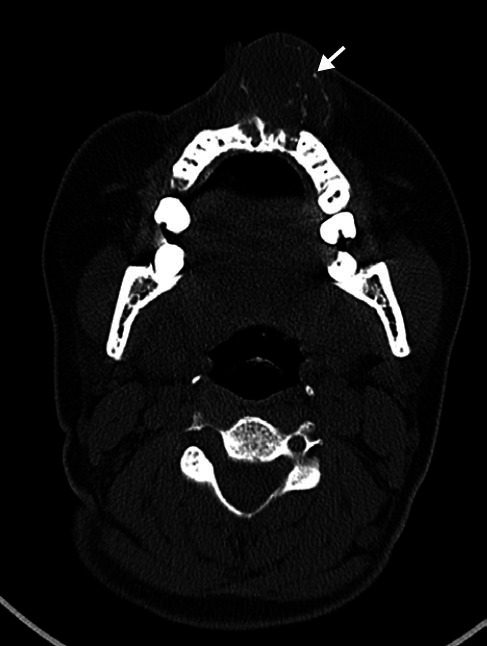
CT scan of an 18-year-old female patient, showing a solid expansile lesion in the maxilla, with areas of fine calcification and a pattern of mineralization indicative of a chondroid matrix (arrow), causing erosion of the maxilla and bulging of the face in the region. The final diagnosis was chondrosarcoma.

Malignant lesions of the mandible and maxilla, although less common^**(2)**^, should always be considered in the differential diagnosis of suspicious radiological findings. Warning signs include extensive lesions, poorly defined margins, an infiltrative pattern, heterogeneous contrast enhancement, central necrosis, aggressive bone destruction, and soft-tissue involvement. These findings indicate the need for histo-pathological investigation and referral to a specialist for determination of the treatment strategy (Figures 17 and 18). Early identification and correlation with the clinical and laboratory findings are essential for proper management.

### Systematic approach to mandibular and maxillary lesions

**Figure 19:**
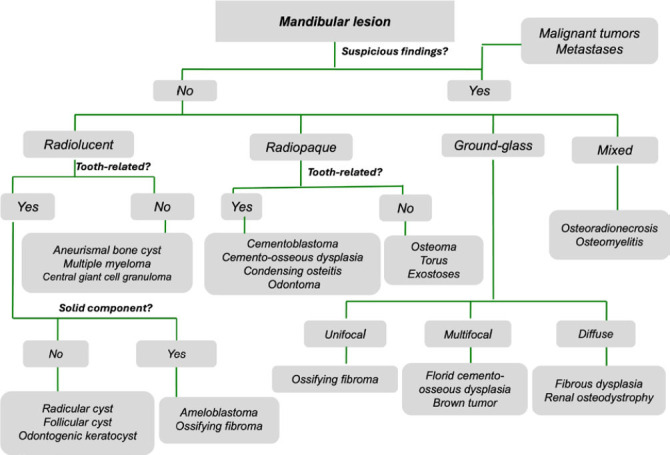
Flow chart of the systematic approach to mandibular lesions based on imaging findings.

Chart 1 summarizes the main radiological findings of the most common lesions of the mandible and maxilla. The analysis should follow a systematic approach (Figure 19), first evaluating signs of aggressiveness—poorly defined margins, infiltrative pattern, heterogeneous enhancement, areas of necrosis, and bone destruction—which suggest malignant tumors, metastases, osteoradionecrosis, or osteomyelitis, especially in lesions with a mixed pattern. In the absence of these signs, the attenuation is classified as radiolucent, radiopaque, or ground-glass. Radiolucent lesions should be evaluated according to their relationship to the teeth: without a solid component, they include radicular cyst, follicular cyst, and odontogenic keratocyst; with a solid component, they include ameloblastoma and ossifying fibroma. If not related to the teeth, they are categorized as aneurysmal bone cysts, multiple myelomas, or central giant cell granulomas. In radiopaque lesions, a relationship with the teeth suggests cementoblastoma, cemento-osseous dysplasia, condensing osteitis, or odontoma; for those not related to the teeth, osteoma, torus, and exostoses are considered. The distribution also facilitates the diagnosis: a unifocal lesion suggests ossifying fibroma; a multifocal lesion suggests florid cemento-osseous dysplasia or a brown tumor. Finally, in lesions with a ground-glass pattern, a unifocal presentation indicates ossifying fibroma, a multifocal presentation indicates florid cemento-osseous dysplasia or a brown tumor, and a diffuse presentation indicates fibrous dysplasia or renal osteodystrophy. Integrating these imaging parameters with clinical and laboratory data allows the radiologist to narrow the differential diagnosis, suggest a management strategy, and, when indicated, recommend biopsy or specialized follow-up.

**Chart 1:** —Main radiological findings characteristic of the most frequent bone lesions of the mandible and maxilla.

Lesion	Imaging finding(s)
Radicular cyst	Radiolucent, unilocular, well-defined, and radicular (< 1 cm), with thin cortical margins and slight bone expansion
Dentigerous cyst	Radiolucent, unilocular, well-defined, surrounding the crown of an impacted tooth, with continuous margins and possible expansion
Odontogenic keratocyst	Radiolucent, unilocular or multilocular, with thin, sclerotic margins, discreet expansion, and root resorption; anteroposterior growth and potential cortical erosion
Ameloblastoma	Expansile, unilocular or multilocular, soap-bubble pattern, root resorption, and cortical thinning; on MRI, solid areas with enhancement and restriction
Ossifying fibroma	Well-defined with sclerotic margins, variable density (lytic, mixed, or ground-glass), and bone remodeling; juvenile variant more aggressive
Aneurysmal bone cyst	Expansile, unilocular or multilocular, with enhancing septa and fluid-fluid levels on CT/MRI; may be associated with preexisting lesions
Multiple myeloma	Radiolucent, ovoid, well-defined, without periosteal expansion or reaction; may exhibit a “punched-out” pattern
Giant cell granuloma	Radiolucent, unilocular or multilocular, expansile tumor with cortical erosion and remodeling; differential diagnosis includes a brown tumor
Odontoma	Radiopaque or mixed, well-defined, with a radiolucent halo; can be compound (tooth-like) or complex (amorphous mass)
Osteoma	Radiopaque, sclerotic, and well-circumscribed, usually pedunculated and unrelated to the teeth; multiple cases suggest Gardner’s syndrome
Torus	Dense, homogeneous, and well-defined cortical exostosis, without halo or expansile effect
Cementum-osseous dysplasia	Initially radiolucent, evolving to ground-glass or radiopacity, with well-defined margins and a multifocal pattern
Brown tumor	Radiolucent, expansile, unilocular or multilocular lesion with cortical thinning and associated diffuse demineralization. Related to hyperparathyroidism
Fibrous dysplasia	Ground-glass, poorly defined margins, infiltrative pattern, and longitudinal growth; can be monostotic or polyostotic
Osteoradionecrosis	Irregular lytic and sclerotic areas, with bone sequestra and cortical interruption
Osteomyelitis	Lytic, sclerotic, or mixed pattern, with chronic periosteal reaction, sequestra, and obliteration of the fat planes; may result from untreated dental infection
Malignant tumors	Extensive, ill-defined margins, infiltrative pattern, necrosis, heterogeneous enhancement, and soft-tissue invasion

### Updates to the WHO 2022 classification and ra-diological implications

The 5th (2022) edition of the WHO classification revisited fundamental concepts that have a direct impact on the radiological interpretation and management of mandibular and maxillary lesions. Of particular note is the regrouping of fibro-osseous lesions into two well-defined categories: ossifying fibroma, now strictly neoplastic and expansile (with a peripheral sclerotic halo and indication for *en bloc* resection), and cemento-osseous dysplasias (radicular, focal, and florid), non-neoplastic processes managed conservatively and characterized by unifocal or multifocal lesions in a ground-glass pattern with well-defined margins. The new classification also returned odontogenic keratocyst to the status of an aggressive cystic lesion, emphasizing, in images, well-defined margins, cortical thinning or erosion, and extension throughout the mandibular canal—characteristics that require surgical planning to include wide margins and prolonged follow-up. New odontogenic carcinomas (such as ameloblastic carcinoma) were also included in the classification, underscoring the need for biopsy whenever soft-tissue infiltration or atypical margins are observed in previously benign lesions. Finally, the 2022 WHO classification reinforced the mandatory clinical–radiological–pathological integration, especially for borderline lesions, as well as refining the definitions of mesenchymal neoplasms, such as ameloblastic fibroma and odontogenic myxoma, so that gelatinous masses and those with infiltrative patterns immediately raise the alert for sarcomatous transformation. These changes provide clearer radiological criteria for distinguishing cysts from tumors, differentiating between hamartomatous and neoplastic processes, and diagnosing malignancies early, ensuring that therapeutic approaches are more precise and effective.

## CONCLUSION

Lesions of the mandible and maxilla, including those that affect the adjacent soft tissue, encompass a wide spectrum of benign, inflammatory, infectious, and malignant entities, often with overlapping radiological features. A systematic approach based on imaging patterns—including attenuation type, margins, relationship with dental structures—is especially useful in cases with atypical presentations or for lesions with overlapping characteristics and anatomical distribution—facilitating diagnostic reasoning and contributing to a more accurate interpretation. Recognizing the signs of aggressiveness and the particularities of each entity allows the radiologist not only to suggest a more precise differential diagnosis but also to act decisively in recommending the appropriate referral and clinical management. In-depth knowledge of these lesions, combined with the clinical and possibly the laboratory correlation, is fundamental in order to avoid diagnostic delays and unfavorable outcomes. The WHO 2022 classification brought major updates regarding the behavior of some odontogenic lesions and the terminology employed to describe them, which underscores the need for continuing education on image interpretation^**(8)**^.

## Data Availability

Not applicable
